# The Olfactory Landscape Concept: A Key Source of Past, Present, and Future Information Driving Animal Movement and Decision-making

**DOI:** 10.1093/biosci/biac039

**Published:** 2022-07-06

**Authors:** Patrick B Finnerty, Clare McArthur, Peter Banks, Catherine Price, Adrian M Shrader

**Affiliations:** Doctor of Philosophy student; University of Sydney, Australia; University of Sydney, Australia; University of Sydney, Australia; University of Pretoria, South Africa

**Keywords:** odor, olfaction, landscape ecology, animal movement, information

## Abstract

Odor is everywhere, emitted across the landscape from predators, prey, decaying carcasses, conspecifics, vegetation, surface water, and smoke. Many animals exploit odor to find food, avoid threats, and attract or judge potential mates. Here, we focus on odor in terrestrial ecosystems to introduce the concept of an olfactory landscape: real-time dynamic olfactory contours reflecting the patchy distribution of resources and risks, providing a key source of information used by many animals in their movement and decision-making. Incorporating the olfactory landscape into current frameworks of movement ecology and animal behavior will provide a mechanistic link to help answer significant questions about where, why, and when many animals move, and how they do so efficiently in both space and time. By understanding how animals use the olfactory landscape to make crucial decisions affecting their fitness, we can then manipulate the landscape to modify ecological interactions and, ultimately, ecosystem consequences of these interactions.

How animals detect and respond to the world  around them has substantial ecological effects beyond individual fitness. Individual movement and decision-making collectively form complex webs of ecologically significant interactions, shaping the structure, dynamics, and evolutionary trajectory of populations, communities, and ecosystems (Nathan et al. 2008, Swingland and Greenwood 1983). Predators exert consumptive and non-consumptive effects on prey (Sheriff et al. 2020, Wirsing et al. 2021), with cascading effects on food webs and nutrient flows within ecosystems (Monk and Schmitz 2021e, Schmitz et al. 2010). Herbivores shape plant communities, affect fire regimes, and impact nutrient recycling processes (Daufresne 2021, Eldridge et al. 2017, Jia et al. 2018, Morgan 2021, Rouet-Leduc et al. 2021, Staver et al. 2021). Tapestries of competitive interactions across environments are formed by individuals moving in response to the presence or absence of con- and hetero-specifics (Forsman and Kivelä 2021, Seppänen et al. 2007). Many other critical ecological services, including pollination and seed dispersal, also often rely on animal movement (Tucker et al. 2021). Consequently, to predict patterns of movement in a landscape by animals to help explain a plethora of ecologically significant interactions, we must first take a step back and understand how animals interact with and navigate their surroundings.

Across a landscape, ‘external factors’ (both the physical environment and living organisms) are key drivers of animal movement and decision making (Nathan et al. 2008). Yet the information and sensory mechanisms animals use to detect and respond to these external factors are seldom discussed. Here we begin by deconstructing current frameworks of animal behavior and movement ecology to highlight a gap in our understanding of the information animals use to make non-random decisions. We argue that odor, as a major information source, and olfaction, as a navigational mechanism, provide a key mechanistic link between how many animal species identify, assess, and respond to their surroundings. We then describe a conceptual theory, the olfactory landscape, elaborate on its unique spatiotemporal flexibility, and argue it as a distinct channel of information allowing early and efficient navigation in many animals. Finally, we discuss manipulating the olfactory landscape to modify ecological interactions and explore potential future directions for its use in conservation and wildlife management.

Although through decades of research, the significance of odor and olfaction in mediating a plethora of ecological interactions has been well demonstrated across both terrestrial and aquatic ecosystems, in this article we will focus on odor in terrestrial ecosystems to develop and demonstrate the olfactory landscape concept.

## Information: a missing link in understanding animal movement and decision-making

Central to current frameworks of animal behavior and movement ecology (Abrahms et al. 2021, Boutin 2018, Gaynor et al. 2019, Lewis et al. 2021, Nathan et al. 2008), decisions to move are largely shaped by four ‘F’ landscapes (Dill 2017): ‘Food’ (e.g. distribution of resources, prey abundance, food quality, surface water availability), ‘Fornication’ (e.g. mating opportunities), ‘Fear and disgust’ (e.g. perceived levels of predation and parasitism risk (Doherty and Ruehle 2020, Laundré et al. 2010, Weinstein et al. 2018)) and ‘Fighting’ (e.g. territoriality and conspecifics). Ultimately, non-random decisions to move require the capacity to sense information about the spatiotemporal distribution of opportunities and threats across these four ‘F’ landscapes (Hein and McKinley 2012). However, the information animals use to detect and respond to these landscapes is rarely considered.

Movement of animals across these four ‘F’ landscapes is intrinsically linked to trade-offs between risk and reward - but how do animals know? Elk feed on lower quality food closer to the safety of the forest when wolves are nearby (Creel et al. 2009), zebra move into lower quality grazing areas and show significant nutritional losses in proximity to lions (Barnier et al. 2014), mandrills avoid parasite contaminated feces and refrain from interacting with infected individuals (Poirotte et al. 2017), elephants move to the greenest areas in the landscape to feed (Loarie et al. 2009), and bushbabies weigh up the relative cost of plant food toxin content and patch safety when foraging (McArthur et al. 2012). But, how do animals locate a foraging patch, or decide that it is or isn't worth visiting from afar? How do animals know when threats move in and out of an area, or when an area is safe to visit? Why do we sometimes observe prey species feeding in typically ‘dangerous’ areas, other than a lack of food in non-dangerous areas? How do animals determine if an individual is infected with parasites or if an area is contaminated? To answer these questions, we require an understanding of the information animals use to make informed decisions.

To date, our understanding of the information animals use to make movement decisions is largely based on physical habitat structure, spatial memory, and visual and audial properties. The importance of physical habitat in providing safety by visual concealment or alternatively in impeding escape (Brown 1992, Ripple and Beschta 2003), and the need for sight lines in animals creating and responding to landscapes of fear have been well documented (Banks et al. 1999, Embar et al. 2011, Shrader et al. 2008, Stears and Shrader 2015, van der Merwe and Brown 2008). Landscapes of sound, or ‘soundscapes’ are recognized as a key information source used in hunting, predator avoidance, foraging, and social communication strategies by many animals (Elmer et al. 2021, O'Connell-Rodwell 2007, Schmidt et al. 2008, Suraci et al. 2019, Suthers 1978). Spatial memory and past experience shape patterns of migration and space use (Fagan et al. 2013, Merkle et al. 2019). Although the importance of odor as an information source and olfaction as a key navigational mechanism has been recognized, this understanding has primarily come from localized studies, focusing on point-sources of information. Consequently, odor at a landscape context as a multilayered and ever-updating information source has been largely overlooked yet is likely fundamental in many animals deciding where to go, and what to eat.

## The olfactory landscape concept

**Figure 1.**
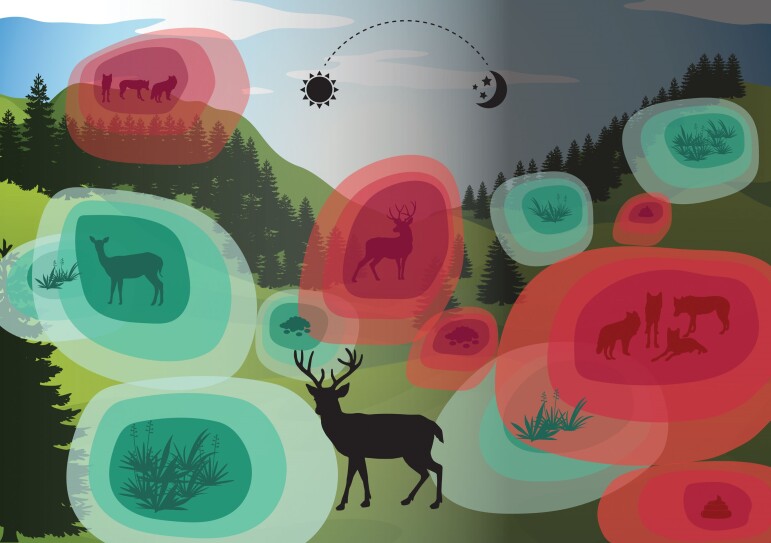
A snapshot of an olfactory landscape. At any one time, an animal is faced with overlapping odor contours emitted from sources of risk (predators, parasitic infected and/or territorial conspecifics) and reward (mating opportunities, food). These olfactory contours are dynamic in space and time, reflecting predator movements, changes in foraging resource quality and location, and territory shifts. For an animal navigating its surroundings, these dynamic contours of odor can be exploited, providing information on the spatiotemporal distribution of potential threats and opportunities. Consequently, across a landscape, these layers of odor provide a key mechanism for many animals to optimize decision making and movement patterns from afar.

Odor is emitted from everything. Odor may be emitted deliberately (e.g. marking territory (Rafiq et al. 2020), signaling sexual state (Marneweck et al. 2017a), as an alarm cue (Joo et al. 2018, Verheggen et al. 2010)), or as an incidental consequence of metabolic processes. Odor also emanates from decaying carcasses (Peterson and Fuentes 2021), from parasite infected matter (e.g. feces) and individuals (Poirotte et al. 2017), from biotic sources such as vegetation (Holopainen and Gershenzon 2010, Šimpraga et al. 2019, Zu et al. 2020), and abiotic sources such as water (Wood et al. 2021), and smoke from fire (Doty et al. 2018, Mendyk et al. 2020, Stawski et al. 2015). Moreover, odor cues may be emitted directly (from the animal, plant, or water source itself) or deposited indirectly (e.g. as a scent mark, fecal matter, or urine). Deliberately emitted or otherwise, odor is everywhere and across the terrestrial landscape dispersion of odor concentrations through wind and air turbulence creates continuously updating contours of olfactory information (Moore and Crimaldi 2004, Riffell Jeffrey A. et al. 2008). As an information source, these dynamic olfactory contours reflect real-time patchy distributions of potential threats and opportunities and are available for all animals to access and then track to a source, ignore, or avoid, given they have the appropriate sensory architecture (figure 1).

There is a plethora of point-based examples of how animals exploit olfactory information. Odor is used to detect and avoid potential threats (Banks et al. 2016, Barnier et al. 2014, Cornhill and Kerley 2020) and locate prey (Hughes et al. 2010). It is used to attract, find and judge mate quality (Harris et al. 2019, Marneweck et al. 2017a, Tirindelli et al. 2009), and recognize conspecifics (Bonadonna and Sanz-Aguilar 2012). It is crucial in marking and maintaining territory (Rafiq et al. 2020, Stępniak et al. 2020) and determining home range distribution (Ranc et al. 2020). Odor has also been shown to be key in locating water (Wood et al. 2021), detecting vegetative food quality and toxicity (Brokaw et al. 2021, Finnerty et al. 2017, McArthur et al. 2019, Schmitt et al. 2018, Skopec et al. 2019) and in detecting and responding to smoke from fire (Doty et al. 2018, Mendyk et al. 2020, Stawski et al. 2015).

**Figure 2.**
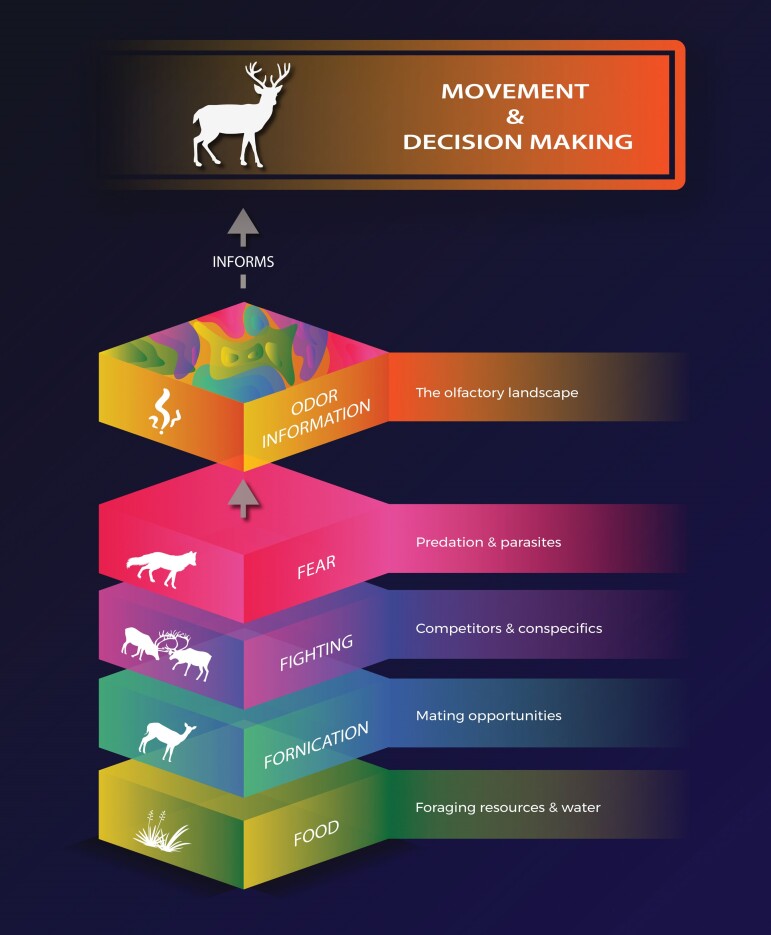
The four ‘F’ landscapes and the olfactory landscape. Current frameworks of animal behaviour and movement ecology focus on two major questions, ‘why move?’ and ‘when and where to move?’. ‘Why move’ is informed by an animal's internal state (e.g., hunger and fear) (Nathan et al. 2008). ‘When and where to move’ is driven by the need to optimize fitness and avoid being eaten across fluctuating external physical landscapes of Food (foraging resources and water), Fornication (mating opportunities), Fighting (competitors and conspecifics), and Fear (predation risk and parasites). But, a missing link in these frameworks between ‘why move’ and ‘when/where to move’ is discussion around ‘how?’. How does an animal know an area is safe to visit? How does an animal decide if an area has food worth moving to? Specifically, the sensory machinery animals use to sense and respond to information, and the information itself comprised of dynamic layers of volatile compounds indicative of resource and risk across these four ‘F’ landscapes is often left out of the equation. Odor is a major source of information answering the ‘how's’ of behaviour and movement for many animals. Across a landscape, dynamic olfactory contours are a consequence of emissions from everything. These dynamic olfactory contours can provide a real-time source of information, reflecting the spatiotemporal distribution of resource and risk across these four ‘F’ landscapes, informing many animals decisions to move.

This growing list of examples provides compelling evidence for the importance of odor as an informative cue, but studies to date typically only allow one dimension of the four ‘F’ landscapes to be examined. All decisions to move involve trade-offs in risk and opportunity, and consequently, these trade-offs cannot be made with only one dimension of information. To detect and respond optimally to ever-changing variation in risk and opportunity in an environment, animals require a regularly updating landscape scale perspective of information (Lima and Bednekoff 1999). By integrating these evidence points of odor as an informative cue, an entire landscape of olfactory information is apparent. Recognizing and incorporating the olfactory landscape into current frameworks of animal behavior and movement ecology will provide a mechanistic link to help answer significant questions about where, why, and when many animals move and respond to ever-changing distributions of resources and risk across the four ‘F’ landscapes, and how they do so efficiently in both space and time (figure 2).

Studies could be designed to better understand the olfactory landscape through two approaches. First, we may eventually be able to map the olfactory landscape as a whole, across the four ‘F's in space and time. Yet, currently available technology continues to remain a key limiting factor in our ability to detect and describe airborne odors (Ivaskovic et al. 2021). In future, as more sophisticated sensory mechanisms are developed, we should be able to detect dynamic waves of olfactory information across landscapes and observe the behaviour and movement of animals responding to it. Second, we can use manipulative experiments to help understand the olfactory landscape and interactive effects of odors across the four Fs. With odor titration studies manipulating odors of one of the ‘F’ landscapes (e.g. adding predator scent cue), we could observe how this effects an animal's interactions with and response to odor information from other ‘F’ landscapes (e.g. herbivore responses to the food plants).

## The olfactory landscape builds upon a distinct information channel

The spatial and temporal dynamics of odor provide information appropriate for early responses in animals. Across a landscape, odor cues can be long lasting and provide information across a range of distances (Celani et al. 2014, Marin et al. 2021, Orlando et al. 2020, Riffell Jeffrey A et al. 2014, Svensson et al. 2014). More mobile volatile components of odor can transport information about food (prey location, vegetation nutritional value, toxicity, surface water availably (Mella et al. 2018, Plotnik et al. 2019, Schmitt et al. 2020, Wood et al. 2021)), threats (predators, competitors, parasites, fire (Poirotte et al. 2017, Valenta et al. 2020) and habitat and reproductive components (kin, mate, and breeding colony location (Caspers et al. 2013, Leclaire et al. 2013, Padget et al. 2017) across potentially large areas. This means animals can receive and interpret this information from afar. Consequently, mobile odor cues enable a highly efficient means of decision-making, well before the odor sources are encountered, with the result that animals can respond early to reduce risk and increase rewards. Moreover, more stationary ‘heavier’ odor compounds can persist in a landscape long after the donor has departed, acting as ‘olfactory billboards’ advertising information left by the donor (Marneweck et al. 2018).

Communal defecation sites (latrines or middens) used by a wide range of mammals including coyote (*Carnis latrans*) (Ralls and Smith 2004), white and black rhino (*Ceratotherium simum* and *Diceros bicornis*) (Linklater Wayne L. et al. 2013, Marneweck et al. 2017a, Marneweck et al. 2018) and oribi antelope (*Ourebia ourebi*) (Brashares and Arcese 1999) are a key example of using such olfactory ‘billboards’. These middens provide multipurpose olfactory communication hubs, advertising an abundance of information about territory, social rank, sex, and oestrus state. Irrespective of whether odor cues are highly mobile or relatively stationary, they degrade and change over time and distance (Marneweck et al. 2017b, Riffell Jeffrey A. et al. 2008, Riffell Jeffrey A et al. 2014). In doing so, odor cues can convey extra information useful in decision making, such as the proximity of an odor source, its age (Bytheway et al. 2013), ripeness (Nevo et al. 2020), or when it was deposited (Cavaggioni et al. 2006).

Consequently, odor can inform and update animals not only about the present, but also about the recent past, and the ‘future’. Because odors linger and spread across the landscape, they can inform animals about the immediate presence and quality of food resources and threats (the present, e.g. predator body odors), on recent threats and resources (the past, e.g. predator or prey urine or conspecific scent marks) and can inform on threats and foraging opportunities from afar (the ‘future’, e.g. the smell of ripe fruit, the odor of predators in the distance) (Nevo et al. 2020). Whether an animal is navigating within a patch, between patches, or across entire landscapes (Senft et al. 1987), odor can therefore provide spatiotemporally dynamic information available for it to exploit when making decisions to move. Moreover, for animals using Bayesian optimization processes to make decisions (Hiratani and Latham 2020), dynamic olfactory landscapes offer ‘live-streaming’ updating of spatial memory ‘maps’, informing them of the past, present, and future distribution of resources and risk across the landscape.

Spatial memory, sight, and sound are other navigational tools, but every sensory system has its limitations. Visual information is curtailed in low light even for nocturnal animals, and fields of view can be obstructed in complex environments. Low frequency sound can travel far (O'Connell-Rodwell 2007), but all sound is momentary and cannot linger in the environment. Spatial memory and past experience may become too outmoded for animals navigating changing landscapes if not regularly updated, and non-existent when they explore novel areas. It is not surprising then, that the spatiotemporal flexibility of the olfactory landscape provides a key complimentary information source and a navigational mechanism that may even dominate other mechanisms and sensory systems for many animals interacting with the world.

## Exploiting the olfactory landscape for novel wildlife management solutions

We can best manipulate animal movement and behavior by understanding the information they use to make decisions. Visual and audial sensory modalities have already been successfully exploited in passively managing and conserving wildlife. For example, ambush carnivore (lions and leopards) attacks on livestock (and subsequent retaliatory killings from landholders) were reduced when ‘eyespots’ were painted on the rump of cattle (McNutt et al. 2017, Radford et al. 2020). Audio playback of matriarchal family groups recordings successfully deter Asian elephants from raiding crops (Larsen and Eigaard 2014, Wijayagunawardane et al. 2016). At finer scales, odor is also used as a management tool. Unpalatable compounds are exploited as repellents and deterrents against herbivorous feeding damage (Gross et al. 2017, Miller et al. 2011, Oniba and Robertson 2019, Sullivan et al. 1988), livestock predation (Smith et al. 2000), and in attempts keep wildlife away from roads and railways (Bíl et al. 2018). Odorous baits are used as lures in attracting problem animals to traps. But, these fine scale solutions produce localized and short-term effects, aiming to stop the behaviors of already motivated animals (Garvey et al. 2020).

From a management perspective, approaches most likely to succeed involve working with animal motivations, rather than against them (Berger-Tal et al. 2011, Garvey et al. 2020, Price et al. 2022). Understanding how to alter an animals’ perceptions of its surroundings (e.g. perceived threats or foraging opportunities) by selectively modifying the information available to an animal would allow for intervention at early stages of movement and decision making processes (Price et al. 2022). In manipulating the motivations and subsequent movement of individuals, we can modify targeted ecological interactions for conservation gain. Recognizing the olfactory landscape as a key information source many animals use to perceive and respond to their surroundings, provides scope to strategically modify this landscape to develop novel conservation approaches.

We are only just beginning to understand how to manipulate the olfactory landscape to alter the motivations of animals for conservation gain. Recent management approaches have shown the effectiveness of exploiting odor to manipulate animal learning and movement patterns. For example, olfactory ‘misinformation’ — unrewarding prey odor cues deployed across a New Zealand landscape — successfully led to a range of invasive predators ignoring ‘unprofitable’ prey cues of two threatened bird species, the South Island pied oystercatcher (*Haematopus finschi*) and the double-banded plover (*Charadrius bicinctus*). These approaches were as effective as lethal control (Norbury et al. 2021).

Manipulating the olfactory landscape to modify targeted ecological interactions provides promising and exciting new pathway for solving current and future conservation and management problems. Prey species reintroduction programs could employ similar olfactory ‘misinformation’ approaches as Price and Banks (2012) to reduce unwanted predation levels on vulnerable prey and also plants. Distributing the scents (e.g. dung, urine) of individuals that are to be re-released into protected areas as part of translocation programs may reduce aggression from resident individuals and promote safe settlement into new areas (Linklater Wayne L et al. 2006, Linklater Wayne L. et al. 2013). Problematic herbivores and granivores could be nudged away from areas of high ecological sensitivity (e.g. revegetation efforts, post fire recovery) or economical value (e.g. agriculture, forestry) by reducing perceived patch palatability (Santiapillai and Read 2010). Alternatively, with landscapes become increasingly fragmented globally, wildlife could be guided towards wildlife corridors, road culverts, and railway bridges using odors, helping to maintain and/or increase landscape connectivity and reduce wildlife mortality rates (Benítez-López et al. 2010, Haddad et al. 2015, Riggio and Caro 2017, Žák et al. 2020).

## Conclusion

As a potential consequence of our own decision making being primarily informed by sight and sound (Atema 1996), we have under-recognized and overlooked the presence of dynamic olfactory landscapes as a key information source used by many animals in deciding where and when to move. The olfactory landscape is an ever-updating information source, reflecting real-time risk and reward distribution. In doing so, it offers a missing link in our understanding of how many animals identify, assess, and respond to patchy external factors they face. Integrating the olfactory landscape into animal behavior and movement ecology frameworks will allow us to better predict patterns of landscape use by animals, helping explain ecologically significant interactions whether predator-prey, plant-herbivore, or between con- and hetero-specifics. Moving forward, we hope that in presenting this conceptual theory we will stimulate new thinking around ways to manipulate the olfactory landscape in developing novel approaches to wildlife and conservation management.

## Author Biographical

Patrick Finnerty (patrick.finnerty@sydney.edu.au) is a Doctor of Philosophy student, Clare McArthur (clare.mcarthur@sydney.edu.au) and Peter Banks (peter.banks@sydney.edu.au) are Professors and Catherine Price (catherine.price@sydney.edu.au) is a Postdoctoral Research Associate in the Behavioural Ecology & Conservation Research Group at the University of Sydney, Australia. Adrian Shrader (adrian.shrader@up.ac.za) is an Associate Professor in the Mammal Research Institute at the University of Pretoria, South Africa.

## References

[bib1] Abrahms B , Aikens EO, Armstrong JB, Deacy WW, Kauffman MJ, Merkle JA. 2021. Emerging Perspectives on Resource Tracking and Animal Movement Ecology. Trends in Ecology & Evolution 36:308–320. 33229137 10.1016/j.tree.2020.10.018

[bib2] Atema J. 1996. Eddy chemotaxis and odor landscapes: exploration of nature with animal sensors. The Biological Bulletin 191:129–138. 29220222 10.2307/1543074

[bib3] Banks PB , Daly A, Bytheway JP. 2016. Predator odours attract other predators, creating an olfactory web of information. Biology Letters 12:20151053. 27194283 10.1098/rsbl.2015.1053 PMC4892236

[bib4] Banks PB , Hume ID, Crowe O. 1999. Behavioural, Morphological and Dietary Response of Rabbits to Predation Risk from Foxes. Oikos 85:247–256.

[bib5] Barnier F , Valeix M, Duncan P, Chamaillé-Jammes S, Barre P, Loveridge AJ, Macdonald DW, Fritz H. 2014. Diet quality in a wild grazer declines under the threat of an ambush predator. Proceedings of the Royal Society B: Biological Sciences 281:20140446. 10.1098/rspb.2014.0446 PMC4024301 24789903

[bib6] Benítez-López A , Alkemade R, Verweij PA. 2010. The impacts of roads and other infrastructure on mammal and bird populations: A meta-analysis. Biological Conservation 143:1307–1316.

[bib7] Berger-Tal O , Polak T, Oron A, Lubin Y, Kotler BP, Saltz D. 2011. Integrating animal behavior and conservation biology: a conceptual framework. Behavioral Ecology 22:236–239.

[bib8] Bíl M , Andrášik R, Bartonička T, Křivánková Z, Sedoník J. 2018. An evaluation of odor repellent effectiveness in prevention of wildlife-vehicle collisions. Journal of Environmental Management 205:209–214. 28987917 10.1016/j.jenvman.2017.09.081

[bib9] Bonadonna F , Sanz-Aguilar A. 2012. Kin recognition and inbreeding avoidance in wild birds: the first evidence for individual kin-related odour recognition. Animal Behaviour 84:509–513.

[bib10] Boutin S. 2018. Hunger makes apex predators do risky things. Journal of Animal Ecology 87:530–532. 29652091 10.1111/1365-2656.12815

[bib11] Brashares JS , Arcese P. 1999. Scent marking in a territorial African antelope: II. The economics of marking with faeces. Animal Behaviour 57:11–17. 10053067 10.1006/anbe.1998.0942

[bib12] Brokaw AF , Davis E, Page RA, Smotherman M. 2021. Flying bats use serial sampling to locate odour sources. Biology Letters 17:20210430. 34665992 10.1098/rsbl.2021.0430 PMC8526173

[bib13] Brown JS. 1992. Patch use under predation risk: I. Models and predictions. Pages 301–309. Annales Zoologici Fennici: JSTOR.

[bib14] Bytheway JP , Carthey AJR, Banks PB. 2013. Risk vs. reward: how predators and prey respond to aging olfactory cues. Behavioral Ecology and Sociobiology 67:715–725.

[bib15] Caspers BA , Hoffman JI, Kohlmeier P, Krüger O, Krause ET. 2013. Olfactory imprinting as a mechanism for nest odour recognition in zebra finches. Animal Behaviour 86:85–90.

[bib16] Cavaggioni A , Mucignat-Caretta C, Redaelli M, Zagotto G. 2006. The scent of urine spots of male mice, Mus musculus: changes in chemical composition over time. Rapid Communications in Mass Spectrometry 20:3741–3746. 17120277 10.1002/rcm.2789

[bib17] Celani A , Villermaux E, Vergassola M. 2014. Odor Landscapes in Turbulent Environments. Physical Review X 4:041015.

[bib18] Cornhill KL , Kerley GIH. 2020. Cheetah behaviour at scent-marking sites indicates differential use by sex and social rank. Ethology 126:976–986.

[bib19] Creel S , Winnie JA, Christianson D. 2009. Glucocorticoid stress hormones and the effect of predation risk on elk reproduction. Proceedings of the National Academy of Sciences 106:12388–12393. 10.1073/pnas.0902235106 PMC2718336 19617549

[bib20] Daufresne T. 2021. A consumer-driven recycling theory for the impact of large herbivores on terrestrial ecosystem stoichiometry. Ecology Letters 24:2598–2610. 34523233 10.1111/ele.13876

[bib21] Dill LM. 2017. Behavioural ecology and marine conservation: a bridge over troubled water? ICES Journal of Marine Science 74:1514–1521.

[bib22] Doherty J-F , Ruehle B. 2020. An Integrated Landscape of Fear and Disgust: The Evolution of Avoidance Behaviors Amidst a Myriad of Natural Enemies. Frontiers in Ecology and Evolution 8.

[bib23] Doty AC , Currie SE, Stawski C, Geiser F. 2018. Can bats sense smoke during deep torpor? Physiology & Behavior 185:31–38. 29253491 10.1016/j.physbeh.2017.12.019

[bib24] Eldridge DJ , Delgado-Baquerizo M, Travers SK, Val J, Oliver I. 2017. Do grazing intensity and herbivore type affect soil health? Insights from a semi-arid productivity gradient. Journal of Applied Ecology 54:976–985.

[bib25] Elmer LK , Madliger CL, Blumstein DT, Elvidge CK, Fernández-Juricic E, Horodysky AZ, Johnson NS, McGuire LP, Swaisgood RR, Cooke SJ. 2021. Exploiting common senses: sensory ecology meets wildlife conservation and management. Conservation Physiology 9. 10.1093/conphys/coab002 PMC8009554 33815799

[bib26] Embar K , Kotler BP, Mukherjee S. 2011. Risk management in optimal foragers: the effect of sightlines and predator type on patch use, time allocation, and vigilance in gerbils. Oikos 120:1657–1666.

[bib27] Fagan WF et al. 2013. Spatial memory and animal movement. Ecology Letters 16:1316–1329. 23953128 10.1111/ele.12165

[bib28] Finnerty PB , Stutz RS, Price CJ, Banks PB, McArthur C. 2017. Leaf odour cues enable non-random foraging by mammalian herbivores. Journal of Animal Ecology 86:1317–1328. 28833142 10.1111/1365-2656.12748

[bib29] Forsman JT , Kivelä SM. 2021. Evolution of searching effort for resources: a missing piece of the puzzle in the ideal free distribution paradigm. Oikos: 08202.

[bib30] Garvey PM et al. 2020. Leveraging Motivations, Personality, and Sensory Cues for Vertebrate Pest Management. Trends in Ecology & Evolution 35: 990–1000. 32900547 10.1016/j.tree.2020.07.007

[bib31] Gaynor KM , Brown JS, Middleton AD, Power ME, Brashasres JS. 2019. Landscapes of Fear: Spatial Patterns of Risk Perception and Response. Trends in Ecology & Evolution 34:355–368. 30745252 10.1016/j.tree.2019.01.004

[bib32] Gross EM , Drouet-Hoguet N, Subedi N, Gross J. 2017. The potential of medicinal and aromatic plants (MAPs) to reduce crop damages by Asian Elephants (Elephas maximus). Crop Protection 100:29–37.

[bib33] Haddad NM et al. 2015. Habitat fragmentation and its lasting impact on Earth's ecosystems. Science Advances 1:e1500052. 26601154 10.1126/sciadv.1500052 PMC4643828

[bib34] Harris RL , Cameron EZ, Nicol SC. 2019. A Field Study of Wild Echidna Responses to Conspecific Odour. Pages 71–80. Cham: Springer International Publishing.

[bib35] Hein AM , McKinley SA. 2012. Sensing and decision-making in random search. Proceedings of the National Academy of Sciences 109:12070–12074. 10.1073/pnas.1202686109 PMC3409737 22778446

[bib36] Hiratani N , Latham PE. 2020. Rapid Bayesian learning in the mammalian olfactory system. Nature Communications 11:3845. 10.1038/s41467-020-17490-0 PMC7395793 32737295

[bib37] Holopainen JK , Gershenzon J. 2010. Multiple stress factors and the emission of plant VOCs. Trends in Plant Science 15:176–184. 20144557 10.1016/j.tplants.2010.01.006

[bib38] Hughes NK , Price CJ, Banks PB. 2010. Predators are attracted to the olfactory signals of prey. PLoS One 5:e13114. 20927352 10.1371/journal.pone.0013114 PMC2948037

[bib39] Ivaskovic P , Ainseba BE, Nicolas Y, Toupance T, Tardy P, Thiéry D. 2021. Sensing of Airborne Infochemicals for Green Pest Management: What Is the Challenge? ACS Sensors 6:3824–3840. 34704740 10.1021/acssensors.1c00917

[bib40] Jia S , Wang X, Yuan Z, Lin F, Ye J, Hao Z, Luskin MS. 2018. Global signal of top-down control of terrestrial plant communities by herbivores. Proceedings of the National Academy of Sciences 115:6237–6242. 10.1073/pnas.1707984115 PMC6004463 29848630

[bib41] Joo Y , Schuman MC, Goldberg JK, Kim S-G, Yon F, Brütting C, Baldwin IT. 2018. Herbivore-induced volatile blends with both “fast” and “slow” components provide robust indirect defence in nature. Functional Ecology 32:136–149.

[bib42] Larsen F , Eigaard OR. 2014. Acoustic alarms reduce bycatch of harbour porpoises in Danish North Sea gillnet fisheries. Fisheries Research 153:108–112.

[bib43] Laundré JW , Hernández L, Ripple WJ. 2010. The landscape of fear: ecological implications of being afraid. The Open Ecology Journal 3.

[bib44] Leclaire S , Nielsen JF, Thavarajah NK, Manser M, Clutton-Brock TH. 2013. Odour-based kin discrimination in the cooperatively breeding meerkat. Biology Letters 9:20121054. 23234867 10.1098/rsbl.2012.1054 PMC3565530

[bib45] Lewis MA , Fagan WF, Auger-Méthé M, Frair J, Fryxell JM, Gros C, Gurarie E, Healy SD, Merkle JA. 2021. Learning and Animal Movement. Frontiers in Ecology and Evolution 9.

[bib46] Lima SL , Bednekoff PA. 1999. Temporal Variation in Danger Drives Antipredator Behavior: The Predation Risk Allocation Hypothesis. The American Naturalist 153:649–659. 10.1086/303202 29585647

[bib47] Linklater WL , Flamand J, Rochat Q, Zekela N, MacDonald E, Swaisgood R, Airton D, Kelly C, Bond K, Schmidt I. 2006. Preliminary analyses of the free-release and scent-broadcasting strategies for black rhinoceros reintroduction. Ecological Journal 7:26–34.

[bib48] Linklater WL , Mayer K, Swaisgood RR. 2013. Chemical signals of age, sex and identity in black rhinoceros. Animal behaviour 85:671–677.

[bib49] Loarie SR , van Aarde RJ, Pimm SL. 2009. Elephant seasonal vegetation preferences across dry and wet savannas. Biological Conservation 142:3099–3107.

[bib50] Marin AC , Schaefer AT, Ackels T. 2021. Spatial information from the odour environment in mammalian olfaction. Cell and Tissue Research 383:473–483. 33515294 10.1007/s00441-020-03395-3 PMC7872987

[bib51] Marneweck C , Jürgens A, Shrader AM. 2017a. Dung odours signal sex, age, territorial and oestrous state in white rhinos. Proceedings of the Royal Society B: Biological Sciences 284:20162376. 10.1098/rspb.2016.2376 PMC5247502 28077775

[bib52] Marneweck C , Jürgens A, Shrader AM. 2017b. Temporal Variation of White Rhino Dung Odours. Journal of Chemical Ecology 43:955–965. 28983753 10.1007/s10886-017-0890-4

[bib53] —. 2018. The role of middens in white rhino olfactory communication. Animal Behaviour 140:7–18.

[bib54] McArthur C , Finnerty PB, Schmitt MH, Shuttleworth A, Shrader AM. 2019. Plant volatiles are a salient cue for foraging mammals: elephants target preferred plants despite background plant odour. Animal behaviour 155:199–216.

[bib55] McArthur C , Orlando P, Banks PB, Brown JS. 2012. The foraging tightrope between predation risk and plant toxins: a matter of concentration. Functional Ecology 26:74–83.

[bib56] McNutt JW , Stein AB, McNutt LB, Jordan NR. 2017. Living on the edge: characteristics of human–wildlife conflict in a traditional livestock community in Botswana. Wildlife Research 44:546–557.

[bib57] Mella VSA , Possell M, Troxell-Smith SM, McArthur C. 2018. Visit, consume and quit: Patch quality affects the three stages of foraging. Journal of Animal Ecology 87:1615–1626. 29995984 10.1111/1365-2656.12882

[bib58] Mendyk RW , Weisse A, Fullerton W. 2020. A wake-up call for sleepy lizards: the olfactory-driven response of Tiliqua rugosa (Reptilia: Squamata: Sauria) to smoke and its implications for fire avoidance behavior. Journal of Ethology 38:161–166.

[bib59] Merkle JA , Sawyer H, Monteith KL, Dwinnell SPH, Fralick GL, Kauffman MJ. 2019. Spatial memory shapes migration and its benefits: evidence from a large herbivore. Ecology Letters 22:1797–1805. 31412429 10.1111/ele.13362

[bib60] Miller AM , O'Reilly-Wapstra JM, Potts BM, McArthur C. 2011. Repellent and stocking guards reduce mammal browsing in eucalypt plantations. New Forests 42:301–316.

[bib61] Monk JD , Schmitz OJ. 2021. Landscapes shaped from the top down: predicting cascading predator effects on spatial biogeochemistry. Oikosn/a.

[bib62] Moore P , Crimaldi J. 2004. Odor landscapes and animal behavior: tracking odor plumes in different physical worlds. Journal of Marine Systems 49:55–64.

[bib63] Morgan JW. 2021. Overabundant native herbivore impacts on native plant communities in south-eastern Australia. Ecological Management & Restoration 22:9–15.

[bib64] Nathan R , Getz WM, Revilla E, Holyoak M, Kadmon R, Saltz D, Smouse PE. 2008. A movement ecology paradigm for unifying organismal movement research. Proceedings of the National Academy of Sciences 105:19052–19059. 10.1073/pnas.0800375105 PMC2614714 19060196

[bib65] Nevo O , Schmitt MH, Ayasse M, Valenta K. 2020. Sweet tooth: Elephants detect fruit sugar levels based on scent alone. Ecology and Evolutionn/a. 10.1002/ece3.6777 PMC7593167 33144973

[bib66] Norbury GL , Price CJ, Latham MC, Brown SJ, Latham ADM, Brownstein GE, Ricardo HC, McArthur NJ, Banks PB. 2021. Misinformation tactics protect rare birds from problem predators. Science Advances 7:eabe4164. 33692107 10.1126/sciadv.abe4164 PMC7946364

[bib67] O'Connell-Rodwell CE. 2007. Keeping an “Ear” to the Ground: Seismic Communication in Elephants. Physiology 22:287–294. 17699882 10.1152/physiol.00008.2007

[bib68] Oniba E , Robertson M. 2019. Trialling a new scent-based repellent to mitigate elephant crop-raiding around Murchison Falls National Park, Uganda. Pachyderm 60:123–125.

[bib69] Orlando CG , Tews A, Banks P, McArthur C. 2020. The power of odour cues in shaping fine-scale search patterns of foraging mammalian herbivores. Biology Letters 16:20200329. 32673541 10.1098/rsbl.2020.0329 PMC7423053

[bib70] Padget O , Dell'Ariccia G, Gagliardo A, González-Solís J, Guilford T. 2017. Anosmia impairs homing orientation but not foraging behaviour in free-ranging shearwaters. Scientific Reports 7:9668. 28851985 10.1038/s41598-017-09738-5 PMC5575321

[bib71] Peterson JV , Fuentes A. 2021. Do long-tailed macaques avoid large heterospecific carcasses? Behaviour 158:341–352.

[bib72] Plotnik JM , Brubaker DL, Dale R, Tiller LN, Mumby HS, Clayton NS. 2019. Elephants have a nose for quantity. Proceedings of the National Academy of Sciences 116:12566–12571. 10.1073/pnas.1818284116 PMC6591706 31160445

[bib73] Poirotte C , Massol F, Herbert A, Willaume E, Bomo PM, Kappeler PM, Charpentier MJE. 2017. Mandrills use olfaction to socially avoid parasitized conspecifics. Science Advances 3:e1601721. 28435875 10.1126/sciadv.1601721 PMC5384805

[bib74] Price C , McArthur C, Norburyb G, Banks P. 2022. Olfactory misinformation: creating ‘fake news’ to reduce problem foraging by wildlife. Frontiers in Ecology and the Environment. doi:10.1002/fee.2534

[bib74a] Price CJ , Banks PB. 2012. Exploiting olfactory learning in alien rats to protect birds’ eggs. Proceedings of the National Academy of Sciences 109:19304–19309. 10.1073/pnas.1210981109 PMC3511072 23071301

[bib75] Radford C , McNutt JW, Rogers T, Maslen B, Jordan N. 2020. Artificial eyespots on cattle reduce predation by large carnivores. Communications Biology 3:430. 32770111 10.1038/s42003-020-01156-0 PMC7414152

[bib76] Rafiq K , Jordan NR, Meloro C, Wilson AM, Hayward MW, Wich SA, McNutt JW. 2020. Scent-marking strategies of a solitary carnivore: boundary and road scent marking in the leopard. Animal behaviour 161:115–126.

[bib77] Ralls K , Smith DA. 2004. Latrine use by san joaquin kit foxes (vulpes macrotis mutica) and coyotes (canis latrans). Western North American Naturalist 64:544–547.

[bib78] Ranc N , Moorcroft PR, Hansen KW, Ossi F, Sforna T, Ferraro E, Brugnoli A, Cagnacci F. 2020. Preference and familiarity mediate spatial responses of a large herbivore to experimental manipulation of resource availability. Scientific Reports 10:11946. 32686691 10.1038/s41598-020-68046-7 PMC7371708

[bib79] Riffell JA , Abrell L, Hildebrand JG. 2008. Physical Processes and Real-Time Chemical Measurement of the Insect Olfactory Environment. Journal of Chemical Ecology 34:837–853. 18548311 10.1007/s10886-008-9490-7 PMC2778261

[bib80] Riffell JA , Shlizerman E, Sanders E, Abrell L, Medina B, Hinterwirth AJ, Kutz JN. 2014. Flower discrimination by pollinators in a dynamic chemical environment. Science 344:1515–1518. 24970087 10.1126/science.1251041

[bib81] Riggio J , Caro T. 2017. Structural connectivity at a national scale: Wildlife corridors in Tanzania. PLoS One 12:e0187407. 29095901 10.1371/journal.pone.0187407 PMC5667852

[bib82] Ripple WJ , Beschta RL. 2003. Wolf reintroduction, predation risk, and cottonwood recovery in Yellowstone National Park. Forest Ecology and Management 184:299–313.

[bib83] Rouet-Leduc J , Pe'er G, Moreira F, Bonn A, Helmer W, Shahsavan Zadeh SAA, Zizka A, van der Plas F. 2021. Effects of large herbivores on fire regimes and wildfire mitigation. Journal of Applied Ecologyn/a.

[bib84] Santiapillai C , Read B. 2010. Would masking the smell of ripening paddy-fields help mitigate human–elephant conflict in Sri Lanka? Oryx 44:509–511.

[bib85] Schmidt KA , Lee E, Ostfeld RS, Sieving K. 2008. Eastern chipmunks increase their perception of predation risk in response to titmouse alarm calls. Behavioral Ecology 19:759–763.

[bib86] Schmitt MH , Shuttleworth A, Shrader AM, Ward D. 2020. The role of volatile plant secondary metabolites as pre-ingestive cues and potential toxins dictating diet selection by African elephants. Oikos 129:24–34.

[bib87] Schmitt MH , Shuttleworth A, Ward D, Shrader AM. 2018. African elephants use plant odours to make foraging decisions across multiple spatial scales. Animal Behaviour 141:17–27.

[bib88] Schmitz OJ , Hawlena D, Trussell GC. 2010. Predator control of ecosystem nutrient dynamics. Ecology Letters 13:1199–1209. 20602626 10.1111/j.1461-0248.2010.01511.x

[bib89] Senft R , Coughenour M, Bailey D, Rittenhouse L, Sala O, Swift D. 1987. Large herbivore foraging and ecological hierarchies. BioScience 37:789–799.

[bib90] Seppänen J-T , Forsman JT, Mönkkönen M, Thomson RL. 2007. Social information use is a process across time, space, and ecology, reaching heterospecifics. Ecology 88:1622–1633. 17645008 10.1890/06-1757.1

[bib91] Sheriff MJ , Peacor SD, Hawlena D, Thaker M. 2020. Non-consumptive predator effects on prey population size: A dearth of evidence. Journal of Animal Ecology 89:1302–1316. 32215909 10.1111/1365-2656.13213

[bib92] Shrader AM , Brown JS, Kerley GIH, Kotler BP. 2008. Do free-ranging domestic goats show ‘landscapes of fear’? Patch use in response to habitat features and predator cues. Journal of Arid Environments 72:1811–1819.

[bib93] Šimpraga M , Ghimire RP, Van Der Straeten D, Blande JD, Kasurinen A, Sorvari J, Holopainen T, Adriaenssens S, Holopainen JK, Kivimäenpää M. 2019. Unravelling the functions of biogenic volatiles in boreal and temperate forest ecosystems. European Journal of Forest Research 138:763–787.

[bib94] Skopec MM , Adams RP, Muir JP. 2019. Terpenes May Serve as Feeding Deterrents and Foraging Cues for Mammalian Herbivores. Journal of Chemical Ecology 45:993–1003. 31755019 10.1007/s10886-019-01117-w

[bib95] Smith ME , Linnell JDC, Odden J, Swenson JE. 2000. Review of methods to reduce livestock depredation II. Aversive conditioning, deterrents and repellents. Acta Agriculturae Scandinavica A: Animal Sciences 50:304–315.

[bib96] Staver AC , Abraham JO, Hempson GP, Karp AT, Faith JT. 2021. The past, present, and future of herbivore impacts on savanna vegetation. Journal of Ecology 109:2804–2822.

[bib97] Stawski C , Matthews JK, Körtner G, Geiser F. 2015. Physiological and behavioural responses of a small heterothermic mammal to fire stimuli. Physiology & Behavior 151:617–622. 26343772 10.1016/j.physbeh.2015.09.002

[bib98] Stears K , Shrader AM. 2015. Increases in food availability can tempt oribi antelope into taking greater risks at both large and small spatial scales. Animal Behaviour 108:155–164.

[bib99] Stępniak KM , Niedźwiecka N, Szewczyk M, Mysłajek RW. 2020. Scent marking in wolves Canis lupus inhabiting managed lowland forests in Poland. Mammal Research 65:629–638.

[bib100] Sullivan TP , Crump DR, Sullivan DS. 1988. Use of predator odors as repellents to reduce feeding damage by herbivores - III. Montane and meadow voles (Microtus montanus and Microtus pennsylvanicus). Journal of Chemical Ecology 14:363–377. 24277015 10.1007/BF01022552

[bib101] Suraci JP , Clinchy M, Zanette LY, Wilmers CC. 2019. Fear of humans as apex predators has landscape-scale impacts from mountain lions to mice. Ecology Letters 22:1578–1586. 31313436 10.1111/ele.13344

[bib102] Suthers RA. 1978. Sensory Ecology of Mammals. Pages 253–287 in Ali MA, ed. Sensory Ecology: Review and Perspectives. Boston, MA: Springer US.

[bib103] Svensson GP , Strandh M, Löfstedt C. 2014. Movements in the olfactory landscape. Animal movement across scales. Oxford University Press, Oxford: 195–218.

[bib104] Swingland IR , Greenwood PJ. 1983. The Ecology of animal movement. Clarendon Press.

[bib105] Tirindelli R , Dibattista M, Pifferi S, Menini A. 2009. From Pheromones to Behavior. Physiological Reviews 89:921–956. 19584317 10.1152/physrev.00037.2008

[bib106] Tucker MA , Busana M, Huijbregts MAJ, Ford AT. 2021. Human-induced reduction in mammalian movements impacts seed dispersal in the tropics. Ecography 44:897–906.

[bib107] Valenta K , Schmitt MH, Ayasse M, Nevo O. 2020. The sensory ecology of fear: African elephants show aversion to olfactory predator signals. Conservation Science and Practicen/a:e306.

[bib108] van der Merwe M , Brown JS. 2008. Mapping the Landscape of Fear of the Cape Ground Squirrel (Xerus inauris). Journal of Mammalogy 89:1162–1169.

[bib109] Verheggen FJ , Haubruge E, Mescher MC. 2010. Alarm pheromones—chemical signaling in response to danger. Pages 215–239. Vitamins & Hormones, vol. 83 Elsevier. 20831948 10.1016/S0083-6729(10)83009-2

[bib110] Weinstein SB , Buck JC, Young HS. 2018. A landscape of disgust. Science 359:1213–1214. 29590062 10.1126/science.aas8694

[bib111] Wijayagunawardane MPB , Short RV, Samarakone TS, Nishany KBM, Harrington H, Perera BVP, Rassool R, Bittner EP. 2016. The use of audio playback to deter crop-raiding Asian elephants. Wildlife Society Bulletin 40:375–379.

[bib112] Wirsing AJ , Heithaus MR, Brown JS, Kotler BP, Schmitz OJ. 2021. The context dependence of non-consumptive predator effects. Ecology Letters 24:113–129. 32990363 10.1111/ele.13614

[bib113] Wood M , Chamaillé-Jammes S, Hammerbacher A, Shrader AM. 2021. African elephants can detect water from natural and artificial sources via olfactory cues. Animal Cognition. 10.1007/s10071-021-01531-2 34292432

[bib114] Žák J , Kraus M, Machová P, Plachý J. 2020. Smart Green Bridge - Wildlife Crossing Bridges of New Generation. IOP Conference Series: Materials Science and Engineering 728:012010.

[bib115] Zu P , Boege K, del-Val E, Schuman MC, Stevenson PC, Zaldivar-Riverón A, Saavedra S. 2020. Information arms race explains plant-herbivore chemical communication in ecological communities. Science 368:1377–1381. 32554595 10.1126/science.aba2965

